# Do different curriculum aligned selection procedures admit students with different personality profiles to medical school?

**DOI:** 10.1371/journal.pone.0209312

**Published:** 2018-12-19

**Authors:** Marieke de Visser, Cornelia Fluit, Janke Cohen-Schotanus, Roland Laan

**Affiliations:** 1 Department for Research in Learning and Education, Radboudumc Health Academy, Radboud University Medical Center, Nijmegen, the Netherlands; 2 Center for Research and Innovation in Medical Education, University of Groningen and University Medical Center Groningen, Groningen, the Netherlands; University of Minho, PORTUGAL

## Abstract

**Background:**

Medical schools aim to contribute to a pool of doctors who are ready for a future practice that will be ever-changing requiring collaboration skills and lifelong learning. They adapt their curricula and selection procedures to fulfil this responsibility. This study aims to determine whether two different selection procedures in one medical school, both matching the key characteristics of the subsequent curricula (one traditional, knowledge-based, and one recently designed for self directed learning and focusing on practice), select students with different personality traits as a side-effect. This perspective was chosen as personality has been related to the CanMeds competencies, innovation capacities, medical school performance and medical professional success.

**Methods:**

A total of 621 students admitted through the new or the traditional selection procedure were invited to complete a Big Five Inventory questionnaire at the start of their Bachelor’s programme. Using ANCOVA, we compared Big Five traits of students admitted through the new selection procedure (n = 196) and the traditional selection procedure (n = 425).

**Results:**

The group of students admitted through the procedure matching the newly designed curriculum had a lower mean score on neuroticism (p < .01) and higher mean scores on conscientiousness, extraversion, agreeableness and openness (p < .001) than the other group.

**Conclusions:**

The findings of the current study indicate that the medical school population is influenced in terms of personality traits as a side-effect of a changing selection procedure. We recommend studying this mechanism and its implications further and using it more consciously in selection procedure design.

## Introduction

Medical schools are the first stage of medical education and, therefore, they have the responsibility to deliver physicians who have the abilities to meet the healthcare needs of the people they serve. Medical schools can do so by designing curricula that favour and enhance the development of the abilities required for future doctors and by attracting and selecting students who either fit into the required profile or have the capacities to fit into it.

The abilities physicians must have to meet the healthcare needs of the people they serve have been defined in the CanMeds model 2015 [1]. This model reflects the profile of the health professional of the future, who is characterized by excellent communication skills, empathy, altruism, integrity and proficiency in teamwork [2–6]. Patients are expected to play a greater role in their healthcare than before (through Shared Decision Making [7], for instance), and collaboration is necessary for healthcare workers to serve patients effectively. Healthcare practice changes constantly and requires reflective and adaptive lifelong learners. Lifelong learning, in turn, requires healthcare professionals to have a clear idea of their own skills, qualities and limitations through constant self-assessment [2–5]. Curricular changes in medical schools aim to prepare students for this future ever-changing practice.

Both the practice and the study of selection have a strong focus on predicting performance in medical school. Research has shown that the strong alignment of selection and curriculum (curriculum sample selection) enhances the admission of students who are likely to perform well in the subsequent curriculum [8–11]. These findings suggest that different curricula and their aligned selection procedures would admit different student populations. Moreover, it raises the question whether a different student population would be admitted if the selection procedure at one medical school were changed to match curriculum adaptations aiming to meet the healthcare needs of the future. Personality is an interesting and relevant angle to explore this question, as it has been related to medical school performance [12–15] and to medical professional success [14, 16]. It has also been related to the CanMeds competencies as mentioned above [17] and to innovation capacities [18]. Therefore, medical schools need to take into account that students’ personality profiles may influence their development to healthcare workers who are ready for future practice.

The aim of the current study was to determine whether two different selection procedures in one medical school, both matching the key characteristics of the subsequent curricula, select students with different personality traits as a side-effect. A selection procedure for a traditional curriculum was compared to a procedure selecting for a curriculum designed to connect more closely into the profile of the healthcare professional of the future [1–5], operationalised through the CanMeds competencies. Personality measures were not included in the selection procedures.

## Methods

### Setting

This study was performed at Radboud University Medical Center (RUMC) in Nijmegen, the Netherlands. The RUMC Bachelor’s curriculum was recently redesigned to connect more closely to the profile of the healthcare professional of the future [1–5], operationalised through the CanMeds competencies. The selection procedure was adapted accordingly. This setting allowed for a research design comparing students admitted through two different selection procedures (traditional versus new), both matching the key characteristics of the subsequent curriculum. In Dutch medical education, a three-year mainly theoretical Bachelor’s programme is generally followed by a three-year Master’s programme with mainly practical education.

### Curricula and admissions

Two different Bachelor’s curricula and the applicable selection procedures were included in the study: the one was applicable before 2015, and the other one has been applicable from 2015 onwards.

#### 2012–2014 selection procedure and curriculum

This selection procedure was a curriculum sample selection as described in greater detail in a previous study [9]. It consisted of an online course followed by an onsite exam. The course and the exam were designed to mimic the curriculum courses and examinations as closely as possible, given the restraints of an online learning environment. The subsequent Bachelor’s programme was launched in 1995 and adapted in 2005. The curriculum was strongly structured and had a strong theoretical approach. It consisted of ten four-week courses in the first and second years, each followed by a summative exam. This system continued in the first part of the third year; in the last part of the third year, students chose from a range of courses during five four-week periods. Furthermore, there was a nursing attachment in year 1 [19] and a practical clinical course in year 3. Each year, students took a professionalism course as well.

#### 2015 selection procedure and curriculum

The selection procedure consisted of two consecutive parts. The first part was given as a home assignment. Applicants were asked to send in a personal description of the Bachelor’s curriculum and their own proficiency. Furthermore, applicants were asked to watch a twelve-minute video showing a general practitioner consultation and to formulate learning objectives and indicate which learning activities they would like to undertake if they had 40 hours available. The second part was an onsite exam consisting of two sections. In the first section applicants were presented with situations and four or five possible actions responding to each situation. They were asked to put the actions in the order of appropriateness to the given situation on the basis of Situational Judgment Test (SJT) principles [20]. The situations represented dilemmas that may occur in the daily practice of medical school or the medical profession and were about working together, giving and receiving feedback, integrity and dealing with mistakes made. The second section was a multiple choice test applying pre-university-level biology, chemistry and physics to medical school issues.

The subsequent Bachelor’s programme, launched in 2015, is characterized by the students’ considerable personal influence on their programme. They can choose learning activities from the programme instead of taking part in obligatory activities. They learn in communities and in patient-centred education from the start onwards. The curriculum is based on the characteristics of the future healthcare professional as described in the Introduction section of this paper, and on the principles of Self-Directed Learning (SDL) and Practice-Based Learning. It is expected that students start to develop SDL through Practice-Based Learning. As the reality of medical professionals changes quickly and constantly, it is important for students to familiarize themselves with (the changes of) everyday care and science practice as soon as possible, to make sure they develop into flexible and adaptive professionals [5]. The ultimate goal of the development of SDL is that students learn to practise the flexibility and autonomy that will be expected from them in their future occupation. Having the ability to set their own goals is expected to enhance students’ motivation for learning [21].

### Admission routes

Each year, 330 students are admitted to the RUMC medical school. In the Netherlands, students have direct access to medical school if their pre-university Grade Point Average (pu-GPA) is equal to or higher than 8 on a scale of 1 (poor)-10 (excellent). This is a national procedure by law. Compulsory subjects included are Dutch, English, Biology, Physics and Chemistry. Mathematics is a compulsory subject as well but is offered in different variations. Other subjects depend on students’ personal choices.

In 2012, students could be admitted to the RUMC medical school through a lottery system in addition to high pu-GPA and selection admissions. This national lottery admission has been described in a previous study [9]. Furthermore, in addition to the selection procedure described above, another selection procedure was also applicable in 2013 and 2014 at RUMC. This was a mainly non-cognitive procedure, not resembling the early medical school curriculum, which was fairly cognitive. In 2012, half the capacity of 330 was available for selection admissions and the other half for lottery and high pu-GPA admissions. In 2013, 2014 and 2015 applicants could be admitted to the RUMC medical school through selection or a high pu-GPA only. The capacity available for selection admissions was 330 minus high pu-GPA admissions.

### Population

A total of 621 students who had been admitted through selection and had enrolled in their medical Bachelor’s programme at the RUMC in September 2012, 2013, 2014 or 2015 were included in the study (Table 1). Students admitted through high pu-GPA (n = 160), lottery (only applicable in 2012, n = 107) and the non-cognitive procedure (2013 and 2014, n = 112) were not included as their admission was not based on a route matching the early medical school curriculum, which was an essential condition for answering our research question.

**Table 1:** Descriptives cohorts 2012–2014 and 2015.

	2012–2014	2015	All cohorts
**N**	425	196	621
**% female (N)**	68.0 (289)	70.9 (139)	68.9 (428)
Median age, years*	18.4	18.6	18.5
**Mean pu-GPA (sd)** *	7.0 (.52)	6.8 (.55)	7.0 (.54)

*significant difference on a p<0.001 level

### Measures

To measure personality, we used the Dutch version of the Big Five Inventory questionnaire (BFIq) [22], which assesses the five subscales conscientiousness, extraversion, openness to experience, agreeableness and neuroticism. Conscientiousness relates to dutifulness, accountability and responsibility. Openness to experience is associated with terms such as originality, creativity, independence and a wide range of interests. Neuroticism is associated with nervousness, anxiety and vulnerability [23]. Extraversion is associated with playfulness, spontaneity, flexibility and assertiveness. The fifth trait is agreeableness, also known as altruism, is associated with compassion, friendliness and helpfulness. The BFIq consists of 44 items on a 5-point Likert scale. Examples of items are: I see myself as a person who -has an active imagination, -is a reliable worker, -is easily distracted.

The Cronbach’s α values of the dimensions of the BFI (neuroticism, conscientiousness, extraversion, agreeableness and openness to experience) in our study were .82, .82, .60, .69 and .76 respectively. These alpha values indicate an acceptable to good internal consistency [24].

### Data collection

Students were invited to complete the BFIq during their first week of medical school. In 2012 and 2013, students were presented with a paper version of the BFIq, and in 2014 and 2015 they were invited to complete a web-based version of exactly the same content. All students received an information letter about the study, and all respondents gave informed consent. Participation was voluntary, and participating students received their personal mean scores and the group average on each subscale of the questionnaire. In 2012, students could choose whether to fill in their registration number (anonymous participation). In 2013, 2014 and 2015, participation was only non-anonymous to make sure that data could be linked. Pu-GPA data of the five compulsory subjects were made available by the Ministry of Education. Data for sex and age were collected from the RUMC student administration.

### Ethics

This study was conducted in accordance with the Declaration of Helsinki. The Radboudumc Research Ethics Committee waived approval for the study. All participants gave informed consent. The 2012–2013 cohorts gave written informed consent, the 2014–2015 cohorts had to give their informed consent online, otherwise they could not proceed to the questionnaire itself. Data were treated strictly confidentially and were available for the researchers only. All analyses were conducted anonymously.

### Data analysis

To assess whether there were differences in the mean pu-GPA of the groups, a t-test was done. A Pearson χ^2^ analysis was conducted to analyse whether the percentage of female students in the groups differed. To analyse whether the median age in the groups differed a Mann-Whitney test was done, as testing showed that the distribution of the data was non-normal for this variable.

To analyse whether there were any differences in personality traits between the students in the two curricula, one-way analysis of covariance (ANCOVA) was used. We controlled for secondary school performance (by pu-GPA), as academic performance and personality have been related in previous studies, for instance by Hakimi et al. [25]. Finally, we calculated Cohen’s d for each of the subscales, to evaluate the effect sizes. The Statistical Package for the Social Sciences (SPSS) Windows version 20 was used for the statistical analyses.

## Results

### Descriptives

Descriptive statistics of the group selected through each procedure are shown in Table 1. The percentage of female students did not significantly differ between groups (χ^2^_(1)_ = .53, p = .47). Compared to the traditional curriculum, the students in the new curriculum had a higher median age (*U* = 31063, *z* = -5.1, *p*<0.001) and a lower mean pu-GPA (t_(1, 589)_ = -4.7, p = 001).

### Personality

For all five scales of the BFIq, significant differences were found between the two groups. Adjusted for pu-GPA, students admitted through the new procedure had a lower mean score on neuroticism than students selected through the previous procedure, F_(1, 580)_ = -6.019, p = .01. On the four other scales (conscientiousness, extraversion, agreeableness and openness), the opposite was found. Adjusted for pu-GPA, students selected through the new procedure had higher mean scores than students selected through the previous procedure as follows: Conscientiousness: F_(1, 576)_ = 31.009, p = .001, Extraversion: F_(1, 582)_ = 18.609, p = .001, Agreeableness: F_(1, 577)_ = 14.981, p = .001, Openness: F_(1, 572)_ = 15.009, p = .001 (Table 2).

**Table 2:** Big Five personality scores of students of two admission routes, adjusted for pu-GPA.

	N	Mean score (sd)	F-value	p-value	Cohen’s d
**Neuroticism**			6.019	0.01	-0.3
- Traditional procedure	406	2.72 (.56)			
- New procedure	177	2.55 (.60)			
**Conscientiousness**			31.009	.00	.05
- Traditional procedure	404	3.68 (.54)			
- New procedure	175	3.95 (.54)			
**Extraversion**			18.609	.00	.05
- Traditional procedure	409	3.55 (.43)			
- New procedure	176	3.75 (.42)			
**Agreeableness**			14.981	.00	.04
- Traditional procedure	404	3.87 (.43)			
- New procedure	176	4.03 (.42)			
**Openness**			15.009	.00	.04
- Traditional procedure	403	3.44 (.52)			
- New procedure	172	3.64 (.48)			

Only non-anonymous responses were included. The non-anonymous response percentages within the total included population are 2012–2014: 70% - 2015:66%. Pu-GPA data were available for 93 and 92 per cent of these, respectively.

## Discussion

This study shows that students admitted to RUMC medical school through two different selection procedures have different personality profiles. The findings indicate that the differences are a side-effect of a curriculum sample selection procedure that was adjusted to fit a changing curriculum.

In the current study, the group of students admitted through the procedure matching the newly designed curriculum has a lower mean score on neuroticism and higher mean scores on conscientiousness, extraversion, agreeableness and openness than the group admitted through the procedure matching the traditional curriculum. In other studies, a high score on neuroticism is related to stress and lower satisfaction in the medical career [16], and agreeableness is related to doctors’ communication skills [14]. Studies also relate Big Five personality traits to medical school performance, with a relatively high consensus across studies [12, 13]. What is most clear across studies is that conscientiousness affects students’ academic performance [14]. Openness to new experiences and extraversion, furthermore, also predict medical school performance [15].

Based on the above-mentioned evidence, Schripsema et al. [17] have described in this journal a set of eligible traits for future doctors. They summarize the set as consisting of conscientiousness, openness, agreeableness and extraversion, as well as modest neuroticism. Schripsema and colleagues’ study suggested that selected students have a lower mean on neuroticism and a higher mean on all other subscales, compared to lottery and to high pu-GPA admitted students in the same cohorts. The authors conclude that the selected group has the best fit with the set of traits of successful medical professionals as evident in the literature and policy statements. In keeping with their line of thought, the students admitted to the newly designed curriculum in the current study also have a better fit than the students admitted to the traditional curriculum.

Patterson and Zibarras [18] relate innovation capacities to Conscientiousness (negative correlation) and Openness to Experience (positive correlation), and they observe that their results indicate that innovation capacities require emotional stability (low Neuroticism). When we reflect on the results found in the current study from this perspective, a mixed picture arises. The newly selected students have a lower score on neuroticism and a higher score on openness, both of which may contribute to innovation capacities. They also have a higher score on conscientiousness, which may have adverse effects on innovation capacities. Based on these data, therefore, we cannot draw a clear conclusion on the innovation capacities of the student population although it is commonly accepted that innovation capacities are crucial for healthcare workers of the future [18].

In interpreting the above relations of personality and communication skills, medical school performance, and innovation capacities, as well as the results of the current study, it is important to consider ‘trait expression’ [26]. The expression of trait-relevant behaviour varies across contexts, meaning that the same person will show different behaviours in different situations. Traditional personality inventories, such as the BFIq, ignore this and only measure ‘typical behavioural tendencies’. Therefore, they do not necessarily predict what someone will actually do in a certain context. As a consequence, the general predictive validity of BFIq results is limited, and direct personality measurement through these kinds of questionnaires appears to be of limited value and may be inappropriate in selection contexts. The effect of the general traits on behaviour in the context after selection is not evident.

The selection procedures in the current study turned out to serve as implicit or ‘contextualized’ personality measures, which are recommended by Ferguson et al. [26] for their additional predictive value compared to traditional inventories. In addition to ‘bright sides’, furthermore, personality traits may also have their ‘dark sides’ [27]. It is impossible to judge each personality trait in general as being positive or negative, or as helpful or detrimental. For a trait to be very high or very low may be detrimental, even for a trait that is generally considered to be helpful for innovation, for instance. This is one more reason for not including general personality measures in selection and assessment subscales in terms of ‘the higher/lower the score, the better’, but for using context-specific measures if you would deliberately include personality in selection.

Our findings raise the question whether the differences between the groups can be explained through the respective curricula, the preceding selection procedures, or both. The strong alignment of the curriculum and the preceding selection procedure is a key characteristic of our selection approach [9], and this, therefore, makes it difficult to unravel the effects. A possible explanation for our findings is that the characteristics of the respective curricula attract different populations. The selection itself, or the type of assignments, may also attract or favour students with certain personality traits: a very structured and cognition-centred approach, for instance, may attract students with high scores on conscientiousness. According to the findings of Schripsema et al., the most plausible explanation is the selection mechanism. They found a different personality profile in selected students than in students admitted to the same curriculum through different admission routes in the same cohorts [17]. We assume, furthermore, that both selection procedures encourage self-selection. Self-selection is a strong and relatively inexpensive mechanism. According to Benbassat and Baumal, medical schools should fully take advantage of self-selection, rather than researching the pros and cons of cognitive and non-cognitive selection and fine-tuning these methods [28].

One could be concerned that indirectly selecting a certain personality profile involves the risk of promoting too much uniformity among medical doctors, applying, as it were, a mould. Like Patterson et al. in their systematic review, we think it is important to critically reflect on selection tools and to consider their risk of limiting the diversity of the student population and future workforce [12]. It seems that each selection procedures in the current study attracts and/or selects a specific personality profile. This may compromise diversity. When we merely focus on diversity of career choice, research shows that there is a loose association between personality factors and particular medical specialties. According to Borges et al., there is a high degree of homogeneity in personality factors across medical specialties [29]. In this respect, the possible concern that having a particular personality profile at the start of the medical education continuum might specifically compromise the diversity of career choices appears to be unwarranted.

### Strengths and limitations

A strength of our study is that it compares consecutive cohorts at one medical school. Both selection procedures match the principles of the curriculum they select for. In a previous study, we showed that curriculum sample selection predicts performance in medical school [9]. This is an important condition in the current study as well, as selection procedures, curricula and the profile of future doctors must be aligned for optimal outcomes.

A limitation of our study is that data were collected through self-report procedures [30], which may limit validity. However, as both groups in the comparison were in similar circumstances, we assume that the potential self-report bias was equal across groups as well. Furthermore, students were not asked to fill in the questionnaires in a high-stakes situation, which might yield invalid or unreliable selection outcomes due to socially desirable responding [31] and ‘faking good behaviour’ [32]. The effect sizes found in our study are moderate (conscientiousness and extraversion) to small (three other subscales) [33]. Conclusions should be drawn with caution, as the differences may not necessarily have an important effect on daily practice. Also, the sample sizes in the groups are different. This may have caused an increased risk of a type I error. Furthermore, the real performance of students in both groups in medical school is what matters most, but this performance cannot be meaningfully compared as the curricula are unequal. While personality was previously considered stable over time [26, 34], there is also a growing body of evidence now on the changeability of personality traits. Therefore, we cannot draw long-term conclusions on the basis of BFIq results.

### Implications for practice

Our results indicate that our selection procedures serve as an implicit measure for certain personality traits. Medical schools may reflect on this mechanism more consciously in their designs, discussing whether it is acceptable to have this implicit measure of personality or whether it is considered a side-effect that should be ruled out. We believe that the evidence about personality traits referred to in the Introduction section of this study, the recent recommendation of Ferguson and Lievens [26] about the context specificity of personality measurement, and the strengths of curriculum sample selection suggest that indirect selection of personality traits through curriculum samples is not a problematic side-effect. It is essential that the selection is a close match of the curriculum, which, in its turn, prepares for future practice. Also, selection procedures should use assessment tools that have proven their predictive value in measuring the key samples of the curriculum [35]. As long as these conditions are met, implicit selection of personality traits is not troublesome.

Irrespective of the outcomes of this discussion and the pros and cons of implicit selection of personality traits, moreover, direct personality assessment through questionnaires may be a useful tool in a learning environment. It is unclear as yet how personality changes as a function of medical training [26], but personality assessment results can be used for student guidance and counselling throughout the academic career [29, 36, 37]. In the current study, students were only given the results of the questionnaire. Additionally they could explicitly be encouraged to understand and implement the results of the questionnaire in a personal plan. This should support them in gaining a better understanding of their strengths, setting learning goals where applicable and reflecting on their progress with a coach.

### Further research

We cannot claim causal relations in this study. The design did not allow us to compare personality profiles of applicants who were rejected and those who were admitted in either procedure. Doing so would help us gain more insight into the autonomous influence of the selection itself ‒ and that of the applicant population that is attracted to it ‒ on the composition of the selected group in terms of personality. Moreover, the current study has been conducted at one medical school. Replication with larger sample sizes in different contexts would give more insight in the pattern found and its generalisability. Nevertheless, we suggest to focus on contextualized trait expression, rather than general BFI measures.

### Conclusions

The findings of the current study indicate that a changing selection procedure has a side effect in terms of differences in personality profiles of the medical school population. We recommend studying this mechanism and its implications further and using it more consciously in selection procedure design.

## Supporting information

S1 File Study data. (XLSX)
